# Defining and Addressing the Current Unmet Medical Needs for the Frontline Treatment of Advanced Stage Aggressive Large B‐Cell Lymphoma: A Perspective From an Ad Hoc Panel of Italian Experts

**DOI:** 10.1002/hon.70152

**Published:** 2025-11-03

**Authors:** Antonio Pinto, Carmelo Carlo‐Stella, Monia Marchetti, Caterina Patti, Annalisa Arcari, Nicola Di Renzo, Marco Laddetto, Maurizio Martelli, Pier Luigi Zinzani

**Affiliations:** ^1^ Hematology‐Oncology and Stem Cell Transplantation Unit Istituto Nazionale Tumori‐IRCCS‐Fondazione ‘G. Pascale’ Naples Italy; ^2^ Department of Biomedical Sciences Humanitas University Milano Italy; ^3^ Department of Oncology and Hematology IRCCS Humanitas Research Hospital Milano Italy; ^4^ Hematology and Transplant Unit Alessandria University Hospital Alessandria Italy; ^5^ Oncohematology Unit Azienda Villa Sofia Cervello Palermo Italy; ^6^ Hematology Unit Ospedale Guglielmo da Saliceto Azienda USL di Piacenza Piacenza Italy; ^7^ Department of Oncology and Hematology Hematology and Stem Cell Transplant Unit. PO “Vito Fazzi” Lecce Italy; ^8^ Haematology Division Università del Piemonte Orientale Azienda Ospedaliera Santi Antonio e Biagio e Cesare Arrigo Alessandria Italy; ^9^ Department of Translational and Precision Medicine Hematology University of Sapienza Roma Rome Italy; ^10^ Department of Medicine and Surgery Bologna University Bologna Italy; ^11^ IRCCS IRCCS Azienda Ospedaliero‐Universitaria di Bologna Istituto di Ematologia “Seràgnoli” Bologna Italy

**Keywords:** advanced stage, diffuse large B‐cell lymphomas, high grade, large, unmet needs

## Abstract

Aggressive large B‐cell lymphomas (LBCL) include a range of disease types characterized by heterogenous histopathologic, molecular, and genetic features. In this review, we summarize the main standardized disease assessments, treatments and patient journey and we discuss some of the questions that we will face in interpreting and applying their results in clinical practice in the next few years. Specific methodologies borrowed from FDA clinical trials indications to settle the more important goals (both for fit and unfit patients) ‐ key aspects to be considered in clinical management—were used. The Expert Panel was conceived to reach a consensus on defining unsettled and controversial issues while envisioning possible solutions for current challenges in treating newly diagnosed patients with advanced‐stage LBCL not only in the Italian context but also for other Countries sharing similar health care system. The fast‐evolving treatment scenario for LBCL holds promise of improving outcomes in these high‐risk setting, and we eagerly await new treatment regimens to further optimize patient outcomes.

## 1 Introduction

Aggressive large B‐cell lymphomas (LBCL) include a range of disease types characterized by heterogenous histopathologic, molecular, and genetic features [1, 2]. Current classification systems, despite some minor taxonomic differences, primarily recognize two main types of disease: diffuse LBCL (DLBCL) and high‐grade B‐cell lymphoma (HGBL). These categories encompass the majority of patients diagnosed with clinically aggressive non‐Hodgkin lymphoma (NHL) originating from mature B‐cells [1, 2, 3].

Worldwide, DLBCL represents the most common subtype of LBCL, accounting for 30%–40% of all newly diagnosed cases of NHL [2]. DLBCL typically presents with aggressive behavior, evolving over months and resulting in symptomatic disease that would imminently be fatal without treatment. Due to recent treatment advances, a greater number of newly diagnosed DLBCL patients can now achieve a cure with improved frontline therapy. Differently, the optimal treatment strategy for patients with HGBL remains largely undefined. This is partly due to the challenging diagnostic definition of this heterogeneous group of entities and the unique and complex molecular genetics of the disease [4].

The clinical and biological heterogeneity of LBCL has been increasingly revealed due to the application of various high‐throughput analytical techniques, many of which, however, are not yet readily available for routine clinical use [1, 2, 3, 5]. Early gene‐expression studies identified two main patient subgroups based on the cell of origin (COO): germinal center B‐cell‐like (GCB) and activated B‐cell‐like (ABC) DLBCL, exhibiting differences in prognosis, with a subset (15%–20%) of unclassified DLBCL [6, 7, 8, 9]. The COO is commonly determined in routine practice using immunohistochemistry (IHC) and the Hans algorithm that categorize patients into the dichotomic subgroups of GCB and non‐GCB cases but may misclassify 10%–20% of patients [10]. For a more accurate definition of the COO, the use of nanostring‐based gene expression profiling (GEP) technology is necessary, but this technology is not routinely available at several Centers [11].

Studies utilizing mutational profiling and genetic data have identified 5 to 7 molecular subsets of LBCL, offering a more precise characterization of the biological diversity within this disease [12, 13, 14, 15]. More recently, Chapuy et al., developed a neural network–based probabilistic classifier, DLBclass, which assigns each DLBCL to its respective C1–C5 genetic subtype prospectively providing a robust classification for single cases inclusion in genetically guided trials or even clinical practice [16].

The intertwined biological complexity of LBCL taxonomies, as emerging by the multilevel analysis of tumor cells (COO, mutational profiling, structural genetic abnormalities, transcriptomics, and proteomics) represents a significant challenge in translating these discoveries into clinically and therapeutically actionable information. While this emphasizes the need for an integrative approach, the ongoing use of techniques involving formalin‐fixed and paraffin‐embedded (FFPE) tumor tissues may streamline this process.

The recent discovery of a unifying gene expression signature, referred to as the “Dark Zone signature” (DZsig), may serve as a tool to identify high‐risk patients with highly aggressive LBCL subtypes who are more likely to benefit from intensified treatments and innovative therapies from the outset [17, 18]. In addition, the DZsig may help in identifying LBCL patients at higher risk for central nervous system (CNS) involvement, possibly contributing to the integration of CNS‐directed treatments in the frontline regimens adopted [4, 19].

Finding a balance between the essential molecular diagnostic requirements for a clinically accurate diagnosis of specific LBCL subtypes and a timely and efficient daily practice remains therefore a current crucial need. This is also because the time from diagnosis to treatment start is a crucial prognostic factor for patients with LBCL [20, 21, 22].

The combination of rituximab with cyclophosphamide, doxorubicin, vincristine, and prednisone (R‐CHOP) has been the gold standard frontline treatment for most patients with LBCL, and specifically DLBCL.

However, approximately 40%–45% of patients treated with R‐CHOP relapse or develop a treatment‐refractory disease [23]. Patients who fail first‐line treatment, particularly those with primary refractory disease, have a very poor prognosis [24, 25]. Therefore, frontline treatments lowering the rate of early failures observed with R‐CHOP should be implemented whenever feasible. Additionally, upfront experimental therapies should be preferred for high‐risk LBCL patients if available through clinical trials. In case of early primary treatment failure, patients should be planned for timely interventions with the most effective salvage strategies, including chimeric antigen receptor (CAR) T‐cells [26, 27]. The accurate definition and early identification of primary refractoriness, along with timely application of the best available subsequent treatments, are then essential needs for the overall strategic planning in the context of LBCL.

Despite the continuous updates to international guidelines, several unmet clinical needs for optimal management of patients with LBCL remain to be fully addressed [28].

This Expert Panel was conceived to reach a consensus on defining unsettled and controversial issues while envisioning possible solutions for current challenges in treating newly diagnosed patients with advanced‐stage LBCL not only in the Italian context but also for other Countries sharing a similar health care system.

## 2 Patients and Methods

### 2.1 Panel Composition

An Expert Panel was formed with 9 members including 2 project leaders and 3 main referrals, of whom one was a methodologist. Both the *Nominal Group Technique* (NGT) and the *Core Outcome Setting* (COS) methodologies were at the basis of the work. The Panel consisted of experienced hematologists from different Italian geographical representations, as showed by their publications, role in clinical trials relevant to the present issues, and involvement in national and international lymphoma associations with also task of guidelines writing. Their expertise covered a broad spectrum of clinical and research perspectives, reflecting real‐world clinical practice in various regions of Italy, encompassing the northern, central, and southern areas of the Country.

### 2.2 Consensus Methodology and Aims

The two project leaders set firstly the main topics: definition of minimal requirements for diagnosis along with the settlement of institutional clinical pathways; treatment goals and choice considering also patients subgroups (hierarchy among endpoints, rank toxicity/feasibility predictors or hurdles); therapeutic algorithm and timing of disease assessment at the various timepoints (interim, end of treatment [EOT] and follow‐up). The Experts met 5 times at virtual conferences to discuss consensus statements and unmet needs in newly diagnosed advanced stage DLBCL to cover the whole patient journey from diagnosis, passing through treatment algorithm to follow‐up at both clinical and logistic level. At the final meeting, The Panel was asked to rate each item related to organizational and logistical diagnostic needs on a 1–5 Likert‐style scale (1 = not important, 5 = very important).

To standardize the set and definition of outcomes for accelerating clinical research and drug development and usage, FDA encourages the development of COS, which represent an agreed minimum set of outcomes that should be measured and reported in all clinical trials of a specific disease or trial population that can be applicable also to everyday clinical practice. To settle hierarchy among endpoints and rank toxicity/feasibility predictors or hurdles the COS was used in this consensus based on the COMET methodology [29].

Panelists were asked to analyze how standard trial outcomes (namely progression‐free survival [PFS], overall survival [OS], event‐free survival [EFS], grade ≥ 3 adverse events [AE] and quality of life [QoL]) were relevant by matching each outcome to each relevance dimension. Each outcome was therefore assigned a Sum Score (SS) and a Relevance Percentage (RP) based on the Likert scores assigned by the Panelists to each outcome‐domain pair. The SS was estimated by assigning weights to of 2, 1, or 0 to Likert scores of 6–6, 4–5, or 1‐3, respectively. RP was estimated by assessing the percentage of clinicians assigning a 6–7 Likert score. Cumulative values across the 6 dimensions were then calculated and outcomes could therefore be ranked into 3 classes. Class 1 included the outcomes summarizing a score higher than 50 or relevance percentage higher than 500%. Class 2 included those outcomes achieving sum‐up scores ranging from 40 to 50 or relevance percentage higher than 400%. Class 3 included those outcomes not adhering to either class 1 or class 2 criteria. Percentages were right after normalized at 100% for more clearness. For further details please see Supplementary Methods.

## 3 Results

**FIGURE 1:**
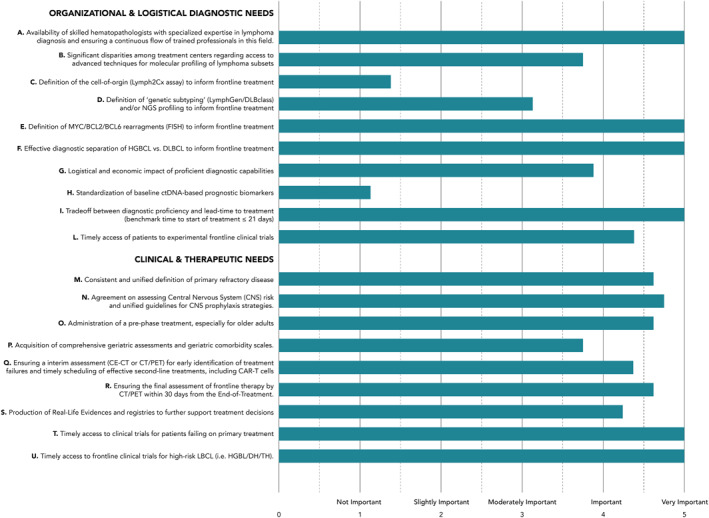
Results of this first round assessment.

In the first instance, Panel members were asked to rate the importance of the main topics and subtopics proposed by the two project leaders. The assessment was based on their personal expertise and geographical location in Italy. The topics covered significant areas related to *organizational, logistical, and diagnostic needs*, as well as *clinical and therapeutic requirements.* Results of this first round assessment are summarized in Figure 1. The Panelists then proceeded to assess the hierarchy among clinical endpoints for frontline therapy and rank toxicity and feasibility predictors using the COS technique.

### 3.1 Diagnosis

#### 3.1.1 Acquisition of Diagnostic Tissue Samples

An excisional/incisional biopsy of a disease‐involved lymph node needs to be obtained. Biopsy should be preferably performed in a 2‐deoxy‐2‐[18 F]fluoro‐D‐glucose (FDG)‐avid site (standardized uptake value [SUV] max‐directed) based on a pre‐treatment FDG‐positron emission tomography (PET) assessment. Fine needle aspiration (FNA) remains inadequate, even when enhanced by flow cytometry or other specialized techniques.

A core needle biopsy should only be performed under specific circumstances related to the limited surgical accessibility of the disease site and the safety of the patient. In these cases, a combination of core biopsies (multiple biopsies are preferred) and FNA biopsies is recommended to obtain adequate FFPE samples for IHC and the minimally required molecular assessments [30, 31]. It is essential to note that while a core biopsy typically provides an accurate primary diagnosis, it does not always enable comprehensive molecular characterization of the disease in every case. Additionally, it may not provide sufficient tissue for banking for future biological studies or enrollment in clinical trials. Such studies have significantly enhanced our understanding of LBCL pathogenesis and are crucial for developing innovative therapeutic approaches for patients. If patients are referred based on a prior tentative diagnosis, a re‐biopsy should be performed if the consultation material is non‐diagnostic or insufficient to allow a proper diagnosis. A staging bone marrow biopsy may be considered when a high‐quality diagnostic PET assessment is unavailable, when there is a discordant diagnosis, or when bone marrow involvement is likely to influence the choice of the initial treatment regimen [32].

#### 3.1.2 Minimal Requirements for Histopathologic Assessment and Immunophenotyping

The essential IHC panel must include antigens that can differentiate between B‐ and T/NK malignancies. Antigens to be detected should include CD20, CD3, CD5, CD10, CD21, CD45, BCL2, BCL6, Ki‐67, IRF4/MUM1, MYC, TP53. IHC detection of the CD19 antigen can be omitted at diagnosis but needs to be performed at first and subsequent relapses to allow the most appropriate sequencing of CD19‐directed second‐ and third‐line treatments.

Experts agree that while the definition of the COO by a nanostring assay (Lymph2Cx) remains relevant for research purposes, it currently has limited utility outside clinical trials. Despite the misclassification inherent in the use of current algorithms, COO assessment through IHC, which distinguishes between GCB and non‐GCB types, is routinely available in most medical Centers. This assessment should be considered as one of the risk factors when selecting a more active primary treatment. The overexpression of Myc (≥ 40%) and Bcl2 (≥ 50%) protein by IHC in the absence of FISH identified translocations is usually associated with the ABC phenotype and identifies the so‐called double expressor B‐cell lymphoma (DEL). This pattern of protein expression was reported as associated with a poor prognosis [33, 34] but the use of this phenotypic description has been deemphasized in the latest lymphoma classifications.

#### 3.1.3 Minimal Requirements for Molecular Assessments

From a clinical perspective, molecular characterization of aggressive LBCL must be finalized at the identification of patients who are at a high risk of early clinical failure after standard frontline treatments. The overlapping morphological and genetic characteristics among entities in the heterogeneous group of LBCL necessitate the clinical distinction of HGBCL, not otherwise specified (NOS) from the DLBCL/HGBCL group with MYC and BCL2 rearrangements. To this end minimally required molecular characterization of LBCL must include the detection of structural status of MYC, BCL2 and BCL6 genes to allow a conclusive histopathologic diagnosis (DLBC, NOS versus with MYC and BCL2 rearrangements).

A FISH analysis for MYC and BCL2 and of BCL6 rearrangements if MYC positive by IHC, remains to date mandatory. The routine implementation and timely availability of the DZsig on FFPE tissues could potentially reduce the need for all the structural analyses mentioned above. This is because DZsig could serve as an objective tool to identify upfront patients who are unlikely to respond effectively to standard treatments [17, 18].

#### 3.1.4 Optimal Requirements for Molecular Assessments

The significance of individual gene mutations in LBCL is complex and not fully understood through prospective clinical trials. These mutations are often linked to specific COO subtypes. Moreover, high‐throughput platforms are not available in all medical Centers, and next‐generation sequencing (NGS) analysis can extend the time from diagnosis to treatment, which has been shown to have its own prognostic implications [20, 21, 22]. Whenever timely available, a prognostic panel for LBCL should include mutations involving the following genes: BCL2, CD79B, FOXO1, KLHL6, MYD88, NOTCH1, SGK1, SOCS1, STAT3, STAT6, and TP53. Mutations in these genes and their regulatory genomic regions have been shown to predict clinical outcomes in patients with LBCL receiving R‐CHOP and similar frontline treatments [5, 12, 16]. In addition, obtaining a molecular profiling for FFPE diagnostic material may represent a tool to implement in the near future strategies based on cell‐free tumor DNA (ctDNA)‐based monitoring of molecular measurable residual disease (mMRD) [35, 36].

Based on these considerations the Panel identified the following current unmet needs for optimal diagnostic characterization of LBCL:

- Adequate availability of hematopathologists specifically trained in lymphomas is critical. Their recruitment and training remain a significant unmet need. Efforts should be made to ensure a continuous influx of trained hematopathologists in conjunction with Pathology Societies [37].
- Nanostring‐based technology for the definition of the COO is routinely available at a minority of Centers, whereas IHC‐based COO assessment according to the Hans algorithm is available at most Centers.
- The definition of double‐hit and triple‐hit HGBCL by FISH is still mandatory.
- The nanostring‐defined DZsig is significant for prognosis. Its wider availability should be pursued for treating DZsig+ LBCL patients with more effective frontline regimens or for enrolling into a clinical trial.
- The NGS assay for determining mutational profiling and comprehensive genomics is not yet regarded as a standard care assessment. However, if possible, FFPE materials should be collected and stored for potential identification of targeted therapies and access to clinical trials.
- ctDNA assessment for mMRD detection does not currently represent a standard of care.
- Pre‐treatment CT‐PET scan with indication of the Deauville score (DS) is mandatory. Contrast‐enhanced CT and PET scans can be performed separately at the time of diagnosis as an alternative option [38].
- Central nervous system (CNS) involvement at diagnosis and risk of CNS progression using CNS‐IPI needs to be assessed in patients with high‐risk LBCL subtypes and in those with high‐risk presentation [39]. In these patients baseline cerebro‐spinal fluid assessment (cytology plus flow immunophenotyping) should be performed. In patients with a clinical suspicion of CNS involvement at diagnosis, a contrast‐enhanced magnetic resonance imaging (MRI) of the brain and spinal cord, if clinically indicated, should be obtained.
- Improving (shortening) the time from diagnosis to treatment is crucial for patients with LBCL due to its significant prognostic implications [20, 21, 22].

### 3.2 Pre‐Phase Treatment

The concept of pre‐phase treatment (steroids, very low‐dose chemotherapy to reduce tumor burden and render the patient fit to receive definitive chemotherapy) was first introduced in 2004 by the German lymphoma group in the NHL‐B2 trial [40]. Pre‐phase therapy improved the performance status (PS) and decreased early treatment‐related mortality [41]. Additionally, because healthcare facilities may not be readily accessible to all patients, pre‐phase treatment may act as a means to prevent complications from chemotherapy that could result in early readmissions.

Following these early observations, many prospective studies, such as MinT, RICOVER‐60, and LNH09‐7B, included pre‐phase treatment as an essential component of therapy [42, 43, 44]. The Panel has reached an agreement that the pre‐phase concept should be regarded as a standard practice, particularly for older adults. The Panel emphasized the impact of a prolonged time between diagnosis and treatment as a negative prognostic factor and recommended a maximum of 3 weeks interval in everyday clinical practice, particularly for high‐risk patients and elderly population [20, 21, 22, 45]. No unmet need or disagreement was reported by the Panel for this issue.

### 3.3 Hierarchy Among Endpoints and Rank Toxicity/Feasibility Predictors—COS

**FIGURE 2:**
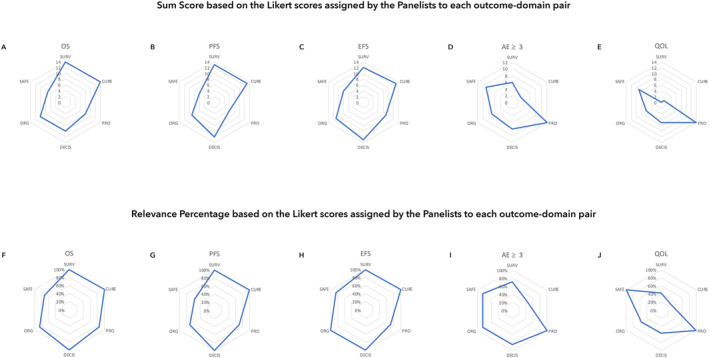
Sum score and relevance percentage based on the likert scores assigned by the panelists to each outcome‐domain pair. A and F, Overall survival (OS); B and G, Progression‐free survival (PFS); C and H, Event‐free survival (EFS); D and I, Adverse event (AE); E and J, Quality of life (QoL). DECIS, decisional impact; ORG, organizational consequences; PRO patient‐reported outcomes; SURV, survival.

No COS is yet available for DLBCL, therefore we chose to select the most relevant outcomes for the treatment of newly diagnosed advanced DLBCL. OS and PFS were the top ranked outcomes according to the survival and cure dimensions: the Relevance Percentage (RP) was 100% and the Sum Score (SS) 12–13, respectively (Figure 2,A,B,F and G). Similarly, the survival outcomes (OS, EFS, PFS) was scored highest regards their decisional (RP 100% SS 10–13) and economic/organizational (RP 71%–100% SS 9–11) impact (Figure 2,A,B,C,F,G and H). However, grade ≥ 3 adverse effect rate was the top ranked outcome according to the patient perspective outcomes (RP 100%, SS 12) and quality of life (QoL) (RP 85%, SS 9) (Figure 2,D,E,I and J).

Cumulative assessment and averaging of SS and RP across the 6 dimensions allowed to rank the outcomes into 2 major classes. Class 1 embraced EFS (mean SS 11, mean RP 92%) and OS (mean SS 10.5, mean RP 90.2%). Class 2 included those outcomes achieving mean SS score ranging from 7 to 10 or mean RP 70%–90%. Four outcomes belonged to class 2, namely PFS (SS 9.8, RP 83%the rate of severe adverse effects (SS 7.5, RP 78%), QoL was scored lowest among the outcomes devoted to patients with DLBCL (SS 6.2, RP 64.2%) and was therefore assigned to class 3.

Composite survival outcomes, particularly EFS, seemed to be the most informative for clinicians managing fit DLBCL patients. EFS provides the most complete overview of the impact that a treatment strategy has in newly diagnosed DLBCL. Rather, any change in patient QoL achieved by a specific therapeutic strategy does not seem to independently support frontline decisions. Surrogate efficacy and safety outcomes, namely CR rate and the rate of severe adverse events, shared a similar informative value.

A second round allowed to specifically select the COS for fit and unfit DLBCL and to assign the 25 clinical outcomes that were listed among primary or secondary endpoints in actively recruiting clinical trials devoted to DLBCL (query to clinicaltrialgov.com performed on February 2^nd^, 2025) to 4 classes.

In the fit patient scenario, the COS was not judged to necessarily include patient‐related outcomes. Rather, EFS, PFS, complete response (CR) rate, CR duration, OS, disease‐free survival (DFS) and treatment‐related mortality were the top ranked outcomes (Summary Weight [SW] 7–10), to be retained in the COS. Overall response rate is not relevant for supporting clinical decisions in fit DLBCL, while response duration might be partially relevant.

In the unfit patient scenario, different outcomes were top ranked (SW 6–7) for inclusion in the COS: EFS, PFS, the rate of treatment‐related severe adverse effects, the rate of severe adverse events, the rate of adverse events overall and the rate of treatment‐related adverse events, as well as treatment‐related mortality. Frailty change granted by the treatment was scored in the second most important outcome class (SW 5).

Overall response rate and MRD were not deems relevant outcomes for inclusion into the trial COS in neither scenario.

Composite survival outcomes, particularly EFS, seemed to be the most informative for clinicians managing fit LBCL patients. EFS provides the most complete overview of the impact that a treatment strategy has in newly diagnosed LBCL. Rather, any change in patient QoL achieved by a specific therapeutic strategy does not seem to independently support frontline decisions. Surrogate efficacy and safety outcomes, namely CR rate and the rate of severe adverse events, shared a similar informative value.

The limited decisional yield of QoL was mostly caused by the limited sensitivity of current QoL assessment tools and the overriding priority of survival endpoints in aggressive diseases such as LBCL. However, the Panelists agreed that QoL data are complementary to survival and safety ones, therefore appropriate composite endpoints should be developed and validated.

### 3.4 Choice of First‐Line Treatment

**FIGURE 3:**
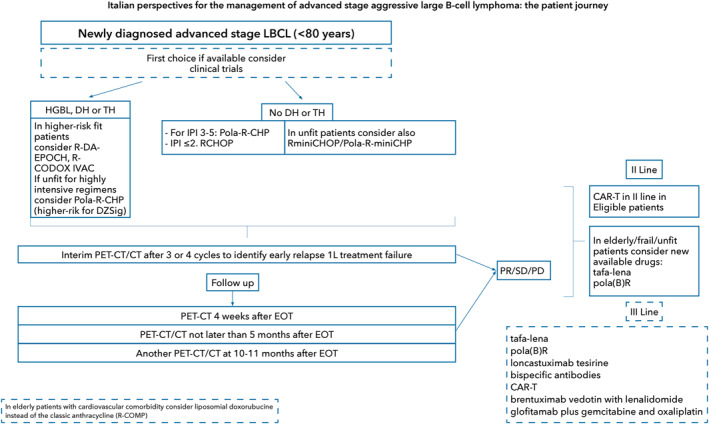
Italian perspectives for the management of aggressive advanced stage large B‐cell patients journey.

For frontline treatment, the Panel recommends enrolling in a clinical trial whenever available, particularly for high‐risk patients based on their primary diagnosis, that is HGBCL, and/or clinical presentation with an IPI> 4. In fit patients with high‐risk histology (HGBL, DH or TH lymphoma), Burkitt Lymphoma (BL)‐like regimens (DA‐EPOCH‐R, R‐CODOX‐M/IVAC) are strongly recommended in case a specific clinical trial is unavailable. For patients with a standard‐risk histology (i.e., non‐HGBL, non‐DH/TH), the Panel recommends a decisional algorithm based on patient fitness, age (e.g., for patients with cardiovascular comorbidity consider to not use anthracycline) and IPI at presentation (Figure 3).

For fit patients with IPI scores of 3–5 and IPI scores of 2 or less, Pola‐R‐CHP and R‐CHOP are respectively recommended. The advantages of Pola‐R‐CHP over R‐CHOP were confirmed by the 5‐year update of the original POLARIX study [46, 47]. Results confirmed a sustained and significant PFS and DFS benefits for patients who received Pola‐R‐CHP versus. R‐CHOP, along with a reduction of lymphoma‐related deaths and a comparable safety profile between the two study arms [46, 47]. In addition, exploratory analyses showed improved 5‐year PFS and OS rates with Pola‐R‐CHP in subgroups with IPI score 3–5 and ABC subtypes by nanostring profiling [48 The PFS advantage over R‐CHOP was even more evident in patients aged ≥ 60 years and, interestingly, in patients with DZSig‐positive disease [48, 49]. Overall, these outcomes confirm Pola‐R‐CHP as a standard of care for the frontline treatment of patients with intermediate‐ or high‐risk DLBCL, including fit older individuals. Preliminary exploratory analyses also suggest that Pola‐R‐CHP may be an option for patients with high‐risk disease biology, specifically those with DZsig‐positive disease who are unable to undergo highly intensive BL‐like regimens [49]. In lower risk patients with IPI ≤ 2, the Panel supports R‐CHOP as a valuable option.

The Panel addressed the issue of unfit patients and older individuals, emphasizing that chronological age should not be an a priori limitation in the strategic planning of treatment aimed at curing disease. In this context, however, the risk of severe treatment‐related toxicity must be weighed against the potential to provide a curative regimen. The panel also highlighted that an objective assessment of the fitness status including comorbidities and main functional inabilities should mandate therapeutic planning [50]. Therefore, in patients aged < 80 years, efforts should be made to propose an active regimen whenever feasible and limit the use of “attenuated regimens” to patients with severe comorbidities. In patients over 80 years old and those younger who are unfit or have moderate comorbidities that do not contraindicate the use of doxorubicin, R‐mini‐CHOP remains a valuable treatment option. Interestingly, a recent real‐life study has demonstrated the efficacy and feasibility of a reduced dosing of the R‐Pola‐CHP regimen in patients with LBCL, prompting further evaluation of this approach [51]. In any case the Panel underlined the importance of the pre‐phase treatment in this patient setting.

The Panel also addressed the use of non‐PEGylated liposomal doxorubicin in the R‐COMP regimen for elderly patients with cardiovascular comorbidity. While the use of the R‐COMP approach is currently limited to the Italian clinical practice, a recent study in patients aged ≥ 65 years concluded that R‐CHOP remains the standard of care in this setting and that R‐COMP may represent a reasonable option only in cases with a significant cardiac comorbidity [52]. The main limitation of the study, however, was the presence of only 22% of patients aged ≥ 80 years in the R‐COMP arm, making it difficult to assess the efficacy/toxicity tradeoff of non‐PEGylated liposomal doxorubicin in this critical age subset. However, panelists were concordant that incoming innovations based on the frontline use of bispecific antibodies and immunomodulatory agents will rapidly modify the current scenario in this setting. The final results of the POLAR‐Bear study, which compares the effectiveness of R‐mini‐CHOP and R‐mini‐CHP with polatuzumab vedotin in patients aged 80 years and older or in frail individuals over 75 years, will provide a clearer understanding of the optimal frontline approach for this growing population of patients [53].

### 3.5 Follow‐Up

#### 3.5.1 Defining Primary Refractory LBCL

The definition of “primary refractory disease” applied to LBCL includes patients who fail to achieve a CR at EOT or who experience early relapse after frontline treatment with anthracycline‐containing immunochemotherapy. Despite being characterized by poor outcomes sustained by refractoriness to chemotherapy, patients with “primary refractory disease” are pretty heterogeneous, as demonstrated by a wide variety of definitions used in the literature and the clinical practice [24, 25, 26, 54, 55, 56, 57, 58, 59, 60, 61]. Such a variety in the definitions of “primary refractory disease” has also emerged in recent trials evaluating CAR T‐cell therapy as second‐line therapy [62, 63, 64]. Also, the use of clinical or pathological predictors to identify primary refractory disease has resulted in conflicting results [64, 65, 66], and the use of different times to treatment failure as inclusion criteria. The SCHOLAR‐1 study, a milestone in the field, defined the refractory status of LBCLs as progressive disease or stable disease as best response at any point during chemotherapy (> 4 cycles of first‐line or 2 cycles of later‐line therapy) or relapsed at ≤ 12 months from autologous stem cell transplantation [25].

Recently, the definition of “primary refractory disease” has been challenged in a study [26] aimed at defining what time to relapse should be used in defining primary refractory disease to identify LBCL patients at the highest risk for poor outcomes to inform clinical practice and future research. Based on this study, patients with stable (SD) or progressive disease (PD) during or at EOT are defined as “primary refractory LBCL”. This is the group of patients with clear chemoresistance and most in need of better treatment options.

Patients with inadequate response or EOT PR (i.e., PR as best response by EOT) and early relapse (i.e., relapse within 12 months) have similar outcomes and may be better grouped as “early relapse”. Both these groups require advanced treatment approaches like CAR T‐cell therapy, bispecific antibodies, or participation in clinical trials due to resistance to approved regimens (Figure 3).

#### 3.5.2 Timing of Interim and Final Response Assessment

Given that the early assessment of disease response is crucial for subsequent therapeutic planning, the Panel recommended to perform a CT‐PET examination after 3–4 cycles of the selected upfront regimen [67], unless the patient is enrolled in a clinical trial with predefined disease evaluation assessment schedule. This will allow timely planning for salvage treatments for primary refractory cases, especially if eligible for second‐line CAR‐T cells or experimental trials.

Final assessment after first line is recommended as a CT/PET scan within 30 days from EOT. If PET‐CT is not readily available, a contrast‐enhanced CT scan may serve as an alternative for EOT assessment. However, if the CT results are uncertain, a PET‐CT scan needs to be obtained. During the follow up after EOT, Panel agreed for a PET within five months after EOT and at 10–11 months after EOT or at clinical suspect for early planning of subsequent treatment (Figure 3).

Based on these considerations the Panel identified the following current unmet needs for timing of interim and final response assessment:

- Consider the difference between early interruption of the first line due to adverse events or intolerance and subsequent progression versus progression only.
- Timing of assessment: consider the feasibility and capability of the hospital (pre‐planning is needed for exam reservation).
- Interim PET results can be considered a prognostic tool for an early shift to second line?
- Which the best score for PET for disease assessment and outcome prediction: DS, SUV o the new emerging metabolic tumor volume (MTV) or total lesion glycolysis (TLG)? [68].
- Timing and type of exams at final assessment after first‐line: consider also central nervous system involvement by contrast‐enhanced magnetic resonance imaging of the brain and/or spinal cord together with cerebrospinal fluid examination (cytology and flow cytometry) especially if CAR T‐therapy is planned.
- Use of methotrexate CNS prophylaxis in patients with relapsing disease (especially for testicular DLBCL) [69].
- Use the recently proposed CNS prognostic model (CNS‐IPI) that includes five IPI factors and the involvement of kidney or adrenal glands [70].

### 3.6 Future Perspectives

Clinical trials devoted to frontline LBCL treatment in fit patients should preferentially target composite outcomes, such as EFS, but should also target the CR rate and, more importantly its quality and duration as expressed by the DFS. Instead, trials devoted to patients who are unfit or not suitable for chemotherapy (including elderly or frail patients) should monitor a different COS, mostly skewed to safety (and indirectly to patient QoL).

This draft fitness‐based COS should be shared with the broader communities of hematologists and other stakeholders, particularly patient representatives, to disseminate the efforts devoted to patient‐related outcomes in specific clinical settings and to translate COS results into daily clinical practice.

At the time of writing, more than 50 trials (phase I‐III) are ongoing, and others are in development, evaluating combinations with newer mono and bispecific antibodies, novel cereblon modifiers, BTK inhibitors, and antibody‐drug conjugates (Table S1). The results of these studies will provide a better understanding of the potential of these novel combinations in newly diagnosed advanced LBCL. Notably, studies evaluating targeted molecular agents *across the board* have not consistently shown a clear benefit, largely due to the biological heterogeneity of the disease. Future research will prioritize specific subsets of LBCL, using novel molecular classifiers for a more personalized approach.

## 4 Conclusions

Management strategies and the treatment landscape for patients with LBCL are continuously evolving, driven by advancements in the molecular classification of this heterogeneous group of clinical and biological entities and the availability of newer active agents. Based on the insights and clinical experiences shared by the Panel of Experts, this consensus provides guidance on the management of challenges in the management of advanced stage LBCL for the frontline approach, with the aim of addressing the unmet needs and maximizing the patients' survival benefits. We have also defined six dimensions as key drivers of unmet medical needs of patients in a given setting. In the absence of quantifiable criteria, different structured expert assessment techniques, can guide patient journey strategies. Recently released international guidelines, including those from the EHA and ESMO, provide objective tools for guiding frontline therapeutic decisions [71, 72]. However, they mirror regulatory scenarios not always readily applicable to all countries and health contexts. The NCCN guidelines, although based on a different clinical and regulatory environment, can serve as a general benchmark for refining treatment strategies in line with the latest registrative trials [28]. Our work focused on the current Italian context reflecting however issues common to other countries with similar NHS‐based therapeutic systems. It is important to note that the main drivers of unmet medical needs for patients with LBCL, as identified in our analysis, align closely with the issues highlighted in the most recent EHA and ESMO guidelines. All these guidelines emphasize the importance of identifying patients at biological risk early, particularly those with HGBL and DH/TH lymphoma. They also highlight a clinical risk‐driven approach (IPI) that favors more active polatuzumab‐containing frontline regimens, the preference, whenever clinically feasible, for more intense (Burkitt lymphoma‐like) treatments in patients with HGBL/DH/TH, and the effort to ensure that older fit patients are not denied access to active frontline regimens solely based on chronological age. The critical need for interim CT‐PET response assessment to timely identify patients who are not responding to upfront treatment, allowing for early referral to effective salvage therapies, was emphasized by the panelists and is reflected in all current guidelines.

Our findings encourage further implementation of the COS methodology and expert‐driven outcome prioritization to better align clinical research endpoints with real‐world decision‐making across various healthcare systems.

In conclusion, by positioning our recommendations within existing international guidelines, we aim to reinforce current standards of care, refine patient selection, and extend clinical practice where evidence is evolving, particularly by defining pragmatic, trial‐ready applications of genomics without advocating routine off‐protocol implementation.

## Author Contributions

A.P. and P.L.Z. conceived the Review. A.P., C.C.S. and P.L.Z. finalized the final draft and supervised the Panel work and contributions. M.M. was the Methodologist. All Authors contributed to the draft of the paper also with pro‐active discussion, edited the draft and approved the final version.

## Funding

Funding was provided by Mattioli Health.

## Conflicts of Interest

C.C.S. received honoraria for consulting/advisory role from ADC Therapeutics, Celgene/BMS, F. Hoffmann‐La Roche, Janssen Oncology, Novartis, SOBI, AstraZeneca, AbbVie, Celgene/BMS, Novartis, SOBI, Merck Sharp & Dohme, Incyte and research funding: ADC Therapeutics, F. Hoffmann‐la Roche. P.L.Z. received honoraria for consulting/advisory role from SOBI, KITE‐GILEAD, JANSSEN, BMS, MSD, AstraZeneca, TAKEDA, F. Hoffmann‐la Roche, RECORDATI, KYOWA KIRIN, Novartis, ADC Therap., Incyte, Beigene. A.A. received honoraria for consulting/advisory role from Hoffmann‐La Roche, Janssen, SOBI, AstraZeneca, AbbVie, Incyte. M.M. received honoraria for consulting/advisory role from GSK, Roche, Alexion, MSD, GSK, Otsuka, Novartis, Roche, Beigene, Menarini. C.P. received honoraria for consulting/advisory role from Abbvie, Astra Zeneca, Janssen, Incyte, Sobi, Roche. N.D.R. received honoraria for consulting/advisory role from, AbbVie, AstraZeneca, Otsuka, Pfizer, Menarini Stemline, Beigene, Celgene/BMS, Incyte. A.P. received honoraria for consulting/advisory role from F. Hoffmann‐La Roche AG, Incyte, Merck, Sharp and Dohme, Kite/Gilead, BMS, BeiGene, Lilly and he is current holder of stock options in a privately‐held company IGM Biosciences, Autolus Therapeutics. M.L. received honoraria for consulting/advisory role AbbVie, Acerta, Amgen, ADC Therapeutics, BeiGene, Celgene/BMS, Eusapharma, GSKI, Gentili, Gilead/Kite, MSD, Novartis, Incyte J&J, Jazz, Lilly, Regeneron, Roche, Sandoz.

## Peer Review

The peer review history for this article is available at https://www.webofscience.com/api/gateway/wos/peer-review/10.1002/hon.70152.

## Supporting information


Supporting Information S1

**Table S1:** Selected ongoing/completed trials for frontline treatment of LBCL.

## Data Availability

Data sharing is not applicable to this article as no new data were created or analyzed in this study.
