# Supplementary material for: Defining and Addressing the Current Unmet Medical Needs for the Frontline Treatment of Advanced Stage Aggressive Large B‐Cell Lymphoma: A Perspective From an Ad Hoc Panel of Italian Experts
Source: Hematol Oncol. 2025 Nov 3;43(6):e70152. doi: 10.1002/hon.70152 (PMC12583570; doi:10.1002/hon.70152)
Supplement: Supplementary file 1 — Supporting Information S1 [file HON-43-e70152-s001.docx]

**Supplementary Methods**

Overall, 6 outcome macro-categories were selected based by the Panelists starting from trial endpoints. Each of the 6 outcomes was assessed by a 7-level Likert scale according to 6 “relevancy” dimensions. The first dimension was “survival” (SURV) since death is the benchmark undesirable outcome for each onco-hematological disease. The second dimension was “cure” since a relevant portion of DBCL can achieve indefinite cure. The third dimension was disease and treatment impact onto patient-reported outcomes (PRO). The fourth dimension was the strategic and “decisional” impact of the outcome for the clinician (DECIS). The fifth dimension was “economic and organizational” consequences (ORG). The last dimension summarized “safety” concerns (grade ≥3 adverse events).

Cumulative values across the 6 dimensions were then calculated and outcomes could therefore be ranked into 3 classes based on the arithmetic mean (51.66) and standard deviation (40.32) of the scores and on the arithmetic mean (471%) of the relevance percentages. Class 1 included the outcomes summarizing a score higher than 50 (arithmetic mean of the sample) or relevance percentage higher than 500% (arithmetic mean of the sample + 10%). Class 2 included those outcomes achieving sum-up scores ranging from 40 (standard deviation of the sample) to 50 or relevance percentage higher than 400% (arithmetic mean of the sample – 10%). Class 3 included those outcomes not adhering to either class 1 or class 2 criteria. Percentages were right after normalized at 100% for more clearness.

Since patient fitness status masters the hierarchy of treatment outcomes, the Panelists were further asked to score the decisional impact of several clinical outcomes in fit and unfit newly diagnosed DLBCL patients. A 1-5 Likert score was assigned to the 25 clinical outcomes that were listed among primary or secondary endpoints in actively recruiting clinical trials devoted to DLBCL (query to clinicaltrialgov.com performed on February 2^nd^, 2025). O*nly actively recruiting phase 3 trials devoted to adult or elderly DLBCL patients were selected.* A summary weight (SW) was estimated by assigning 2 points to Likert score 5 (determinant to the decision), 1 point to Likert score 4 (high decisional impact) and – 1 or -2 points to Likert scores 1 or 2 (poorly relevant), respectively.
