# Supplementary material for: Defining and Addressing the Current Unmet Medical Needs for the Frontline Treatment of Advanced Stage Aggressive Large B‐Cell Lymphoma: A Perspective From an Ad Hoc Panel of Italian Experts
Source: Hematol Oncol. 2025 Nov 3;43(6):e70152. doi: 10.1002/hon.70152 (PMC12583570; doi:10.1002/hon.70152)
Supplement: Supplementary file 2 — Table S1: Selected ongoing/completed trials for frontline treatment of LBCL. [file HON-43-e70152-s002.docx]

Supplementary Table 1. Selected ongoing/completed trials for frontline treatment of LBCL

| Regimen | Agents added to treatment backbone | Enrolled/planned patients |  |
| --- | --- | --- | --- |
| R-CHOP plus tafasitamab-lenalidomide | Anti-CD19 monospecific antibody and IMID | 880 | NCT04824092 |
| R-CHOP plus acalabrutinib | BTK inhibitor | 600 | NCT04529772 |
| R-CHOP plus epcoritamab | CD20xCD3 bispecific antibody | 1100 | NCT05578976 |
| POLA-R-CHP plus glofitamab | Polatuzumab and 2:1 CD20xCD3 bispecific antibody | 750 | NCT06047080 |
| R-CHOP plus golcaldomide (GOLCA; CC-99282) | Cereblon E3 ligase modulator | 900 | NCT04884035 |
| CHOP plus odronextamab | CD20xCD3 bispecific antibody | 840 | NCT06091865 |
| R-CHP plus zilovertamab vedotin | Anti-ROR1 antibody-drug conjugate | 1046 | NCT06717347 |
| R-mini-CHP | Polatuzumab | 200 | NCT04332822 |

Abbreviations: BTK, Bruton’s tyrosine kinase; IMID, immunomodulatory imide drug; POLA, polatuzumab vedotin; R-CHOP, rituximab, cyclophosphamide, doxorubicin vincristine.
